# Direct Electrical Stimulation in Electrocorticographic Brain–Computer Interfaces: Enabling Technologies for Input to Cortex

**DOI:** 10.3389/fnins.2019.00804

**Published:** 2019-08-07

**Authors:** David J. Caldwell, Jeffrey G. Ojemann, Rajesh P. N. Rao

**Affiliations:** ^1^Department of Bioengineering, University of Washington, Seattle, WA, United States; ^2^Medical Scientist Training Program, University of Washington, Seattle, WA, United States; ^3^Center for Neurotechnology, University of Washington, Seattle, WA, United States; ^4^Department of Neurological Surgery, University of Washington, Seattle, WA, United States; ^5^Paul G. Allen School of Computer Science and Engineering, University of Washington, Seattle, WA, United States

**Keywords:** electrocorticography, brain–computer interface (BCI), direct electrical stimulation, intracranial electrodes, plasticity induction, neuroprosthetic, sensory restoration, neuromodulation

## Abstract

Electrocorticographic brain computer interfaces (ECoG-BCIs) offer tremendous opportunities for restoring function in individuals suffering from neurological damage and for advancing basic neuroscience knowledge. ECoG electrodes are already commonly used clinically for monitoring epilepsy and have greater spatial specificity in recording neuronal activity than techniques such as electroencephalography (EEG). Much work to date in the field has focused on using ECoG signals recorded from cortex as control outputs for driving end effectors. An equally important but less explored application of an ECoG-BCI is directing input into cortex using ECoG electrodes for direct electrical stimulation (DES). Combining DES with ECoG recording enables a truly bidirectional BCI, where information is both read from and written to the brain. We discuss the advantages and opportunities, as well as the barriers and challenges presented by using DES in an ECoG-BCI. In this article, we review ECoG electrodes, the physics and physiology of DES, and the use of electrical stimulation of the brain for the clinical treatment of disorders such as epilepsy and Parkinson’s disease. We briefly discuss some of the translational, regulatory, financial, and ethical concerns regarding ECoG-BCIs. Next, we describe the use of ECoG-based DES for providing sensory feedback and for probing and modifying cortical connectivity. We explore future directions, which may draw on invasive animal studies with penetrating and surface electrodes as well as non-invasive stimulation methods such as transcranial magnetic stimulation (TMS). We conclude by describing enabling technologies, such as smaller ECoG electrodes for more precise targeting of cortical areas, signal processing strategies for simultaneous stimulation and recording, and computational modeling and algorithms for tailoring stimulation to each individual brain.

## Introduction

Electrocorticography (ECoG) is used clinically as a recording modality for diagnosing specific spatial regions of focal epilepsy onset in individuals suffering from medically intractable epilepsy. By using invasive monitoring, the origins of seizures can be identified, and subsequent surgical removal of the seizure foci can reduce the frequency of or eliminate seizures. After surgical resection, approximately 50% or greater of patients experience significantly improved seizure control following surgical treatment (Englot and Chang, 2014). For monitoring, patients are routinely implanted for 1–2 weeks with electrodes either directly on top of the dura (epidural), beneath the dura (subdural), or implanted in cortex [depth electrodes, or stereo electroencephalography (sEEG)]. The term intracranial EEG, or iEEG, is often used to describe all implanted electrodes. We will use the term ECoG electrodes in this article to encompass surface as well as penetrating depth electrodes. Following electrode implantation, patients remain in the hospital under clinical monitoring by a team of neurologists and epilepsy technicians, until the clinical team has collected enough data to precisely localize the focal seizure zones for surgical resection.

To complement the passive recording of epileptic events, direct electrical stimulation (DES) (Vincent et al., 2016b) [or when applied particularly to cortex, known as direct cortical stimulation (DCS) (Giussani et al., 2010), or direct electrical cortical stimulation (DECS)] through ECoG electrodes is commonly performed for clinical mapping purposes, both intraoperatively and during the patients’ clinical observation. For clinical mapping the clinical team electrically stimulates different brain regions to delineate regions of cortex important for language, motor, and sensory function. By stimulating particular brain areas and observing the effects by querying the patient, the clinical team can avoid resecting areas important for cognitive function and preserve these functions in an individual after surgical resection. The combination of recording and mapping through stimulation enables the clinical team to be best informed when making clinical decisions regarding reducing or eliminating seizures through resection, while maintaining cortical function. Clinical teams perform stimulation of both cortical and subcortical structures and pathways. We use the term DES here to refer to general electrical stimulation of any brain region through implanted electrodes, while we consider DCS a subcategory specifically describing stimulation of surface gray matter.

Direct electrical stimulation for clinical uses goes beyond delineating cortical regions of activity. For example, deep brain stimulation (DBS) is a therapy currently being used for therapeutic treatment of movement disorders and psychiatric illnesses. Electrodes similar to those used for sEEG are implanted into deep brain structures, and stimulation helps ameliorate clinical symptoms. The space of DBS research is vast, and we will not go into extensive detail in this review. Instead, we highlight the widespread use of DBS as a demonstration of the therapeutic use of clinical stimulation through implanted electrodes, and we draw from current research in the DBS field to frame future directions for DES.

In this article, we first review the characteristics of implanted electrodes, the effect of electrical stimulation through them on cortex, and the nature of signals recorded through them. We then discuss current clinical uses of DES for disorders such as epilepsy, and briefly cover DBS and its applications for diseases such as Parkinson’s disease and essential tremor. We also discuss some of the translational, regulatory, financial, and ethical concerns with ECoG-BCIs. We subsequently describe how ECoG-based DES can be used to provide sensory feedback and to probe and modify cortical connectivity. We then review future applications of DES in ECoG-BCIs, which may draw from invasive animal studies with penetrating and surface electrodes and non-invasive stimulation methods such as transcranial magnetic stimulation (TMS) and transcranial electrical stimulation (TES). We discuss how enabling technologies, such as smaller ECoG electrodes for more precise targeting on smaller spatial scales, software and hardware that allow simultaneous stimulation and recording, and computational modeling for tailoring stimulation to individual patients, could enable the realization of full-fledged bidirectional ECoG-BCIs for a variety of applications.

## The Electrical-Neural Interface

### Electrodes

Current clinically used ECoG electrodes are often embedded in a silicone sheet and are made of platinum or stainless steel. The electrodes are 1.5 mm diameter circular contacts with 4 mm spacing (“micro”-ECoG electrodes)^1^, to 2.3–3 mm diameter contacts with 10 mm spacing for “macro”-ECoG electrodes (Chang, 2015). Depth electrodes are frequently comprised of platinum, with cylindrical contacts, and can be inserted with or without stereotactic guidance. These are commonly used to localize seizures coming from deep brain structures, such as the hippocampus. DBS electrodes are similar to depth electrodes in that they are linear probes with cylindrical contacts, although they can be of smaller diameter, with tighter electrode spacing and fewer contacts.

### Stimulation

Implanted electrodes can be used for direct modulation of neural activity through electrical stimulation. In order to better understand the underlying mechanisms of stimulation, we first consider the effects of stimulation on a single neuron. At the single neuron level, the redistribution of charge, and subsequent depolarization, where the inside of the cell becomes more positive relative to the extracellular fluid, can cause an action potential to be generated which propagates down the cell’s axon. Hyperpolarization, which occurs when the inside of the cell becomes more negative relative to the outside of the cell, can inhibit action potentials. Electrical stimulation, through a redistribution of charge within an axon, can result either in hyperpolarization or depolarization. When sufficient depolarization is achieved, an action potential is generated through the diffusion of ions through sodium, potassium, and calcium channels (Bean, 2007). Subthreshold intracellular stimulation, where an action potential is not generated, can result in the potentiation of synaptic strength with NMDA receptor mediation in the neuron’s synapses (Alonso et al., 1990).

**FIGURE 1:**
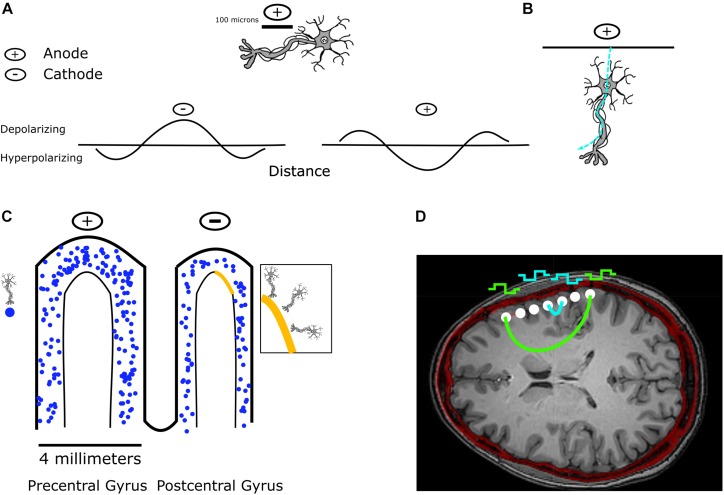
Effect of stimulation on a single neuron and on a population of neurons. **(A)** Stimulation along a nerve fiber results in depolarization beneath the cathode, and hyperpolarization beneath an anode. **(B)** Single neurons can be stimulated by both anodal and cathodal stimulation depending on their orientation. In this example, anodal current enters the dendrites of the neuron and leaves through the axon, which results in depolarization of the axon and an action potential. **(C)** In the case of stimulation through ECoG electrodes, a large population of neurons can be activated by stimulation. Shown are approximate scales of an ECoG electrode relative to the precentral and postcentral gyrus, along with a representative mixed population of pyramidal neurons potentially depolarized by stimulation. In the zoomed-in region, we highlight the multiple orientations of neurons that could be activated. **(D)** An axial slice in a co-registered CT and MRI image following implantation with an ECoG array shows the potential current paths that different stimulation configurations would have to pass through, illustrating the large populations of neurons present within the potential current path. **(A,B)** Inspired by Ranck (1975).

In solutions, electrical stimulation results in the redistribution of ions through non-Faradaic reactions, and the transfer of electrons to electrolytes in the solution through Faradaic reactions (Merrill et al., 2005). There exist both reversible and irreversible Faradaic reactions: which one occurs depends on the rate of the electron transfers relative to the mass transport of the reactant. We discuss these reactions further and the impact of stimulation parameters on them in the section “Limitations and Considerations.” Through these mechanisms, charge is redistributed. When this redistribution of charge causes depolarization directly beneath the electrode, for the case of a single neuron, the stimulation is often referred to as cathodal stimulation, while electrical stimulation which causes hyperpolarization directly beneath the electrode is referred to as anodal stimulation (Figure 1A). On the scale of larger electrodes, such as with ECoG arrays, cathodal stimulation often refers to negative voltages and currents directly beneath the electrode, while anodal stimulation refers to positive voltages and currents.

Stimulation on a local scale can be achieved through intracortical microstimulation (ICMS), where electrical stimulation activates neurons primarily through their axons passing through the region of cortex stimulated (Nowak and Bullier, 1998; Tehovnik et al., 2006). However, other regions of the cell such as the cell body and dendrites may also be activated depending on stimulus polarity and orientation. Anodal pulses best activate cell bodies and terminals, compared to cathodal pulses which best activate axons (McIntyre and Grill, 2000). In both cases, it is the outward flowing current at the axon initial segment or nodes of Ranvier along the axon that results in neuronal excitation (McIntyre and Grill, 2000; Tehovnik et al., 2006). ICMS is thought to sparsely activate a population of cortical neurons, rather than just ones proximal to the stimulation electrode tip (Histed et al., 2009).

The distance of neuron elements from the stimulation source changes whether or not these elements will be hyperpolarized or depolarized by a corresponding cathodic or anodic stimulus. Directly beneath a cathode, a membrane will become depolarized, and hence can generate an action potential. During the case of anodal stimulation, the area directly beneath the electrode is hyperpolarized, but further away from the anode, action potentials may be generated, resulting in a “virtual cathode” (Merrill et al., 2005) (Figure 1A). Stimulation beneath the anode can occur with surface anodal stimulation of neocortical cells, where current hyperpolarizes apical dendrites, and subsequently leaves through the axon resulting in depolarization (Ranck, 1975) (Figure 1B). For the case of bipolar stimulation, an axon is generally depolarized beneath the cathode and hyperpolarized beneath the anode (Ranck, 1975).

Physiologically, ICMS is thought to activate both inhibitory and excitatory populations of cells (Butovas and Schwarz, 2003), and is not thought to evoke natural patterns of cortical activity (Millard et al., 2015). Functional magnetic resonance imaging (fMRI) along with microstimulation has demonstrated that microstimulation, at least in the visual cortical pathway, suppresses the output activity of neurons which have their afferents stimulated (Logothetis et al., 2010). Further work in microstimulation of the visual cortex has demonstrated that microstimulation in V1 may locally activate cells, but silence neurons further downstream (Klink et al., 2017).

The frequency of ICMS also has an impact on whether neurons are excited or inhibited. High frequency stimulation (>10 Hz) is thought to potentiate neural activity (long-term potentiation) (Bliss and Lomo, 1973; Douglas, 1977), while low frequency stimulation (<1 Hz) is thought to depress neural activity (long-term depression) (Mulkey and Malenka, 1992).

Compared to ICMS, DES of human cortex using larger electrodes, such as ECoG or DBS electrodes, injects current over a larger surface area, and subsequently large amounts of current could lead to greater activation with the potential to spread to a larger area (Vincent et al., 2016a) (Figures 1C,D). Additionally, depending on the anatomic location of DES, stimulation can either evoke or inhibit neural activity (Borchers et al., 2012). For example, DES of language areas during a language task can disrupt speech production while DES of somatosensory cortex can evoke sensations and DES of motor cortex can evoke movements. In terms of subdural ECoG stimulation in humans, the patterns and types of cells activated are thought to depend on the intricate details of cortical geometry, cell fiber orientation (Kudela and Anderson, 2015), and whether the pulses are anodal or cathodal (Seo et al., 2015) (Figures 1C,D). A finite element model (FEM) of subdural cortical stimulation with integrated neuron models was used to demonstrate that neurons deeper in the bank (buried in cortex) are more activated during cathodal subdural stimulation, while those in the wider crown are activated during anodal stimulation (Seo et al., 2015). DES through ECoG electrodes can result in both local effects, and effects remote to stimulation. The resultant signals at other electrodes are often referred to as cortico-cortico evoked potentials (CCEP) (Keller et al., 2014b). These have been reviewed thoroughly by Keller et al. (2014b, for more details). We will therefore only review some of the relevant physiology here. Pyramidal cells, which are the source of the majority of cortical output, and lie in cortical layers 2, 3, 5, and 6 can have their superficial dendritic trees depolarized. Layer 2/3 inhibitory GABA interneurons can be depolarized, which then synapse preferentially near the soma of pyramidal cells (Brill and Huguenard, 2009) and cause a decrease in pyramidal cell activity due to the inhibitory nature of GABA signaling. If there are axons passing through the region of stimulation, both orthodromic and antidromic stimulation can occur (Keller et al., 2014b). The measured surface potentials are therefore a combination of the initial monosynaptic connections, cortico-cortical pathways, and cortico-subcortical pathways which would explain the polymorphic response lasting hundreds of milliseconds (Matsumoto et al., 2006).

The mechanisms of DBS stimulation are not yet currently understood, and are thought to involve the modulation of the networks targeted by the stimulation, rather than involving solely immediate inhibitory effects on the targeted anatomic region (Montgomery and Gale, 2008; Ashkan et al., 2017).

In summary, the results reviewed in this section speak to the immense complexities of engineering stimulation in humans and the work that remains to be done in understanding both its physical and neural effects.

### Sensing

A key part of a BCI is the recording of neural activity to use as a control signal in order to successfully modulate the system using stimulation. The summed activity of many hundreds of thousands of neurons in the cortical tissue under an ECoG electrode contributes to the electric voltage recorded from the electrode. The increased firing rate of populations of neurons results in a broad increase in power across all frequencies, which is more easily separable in the broadband gamma band (above 50 Hz), rather than the lower frequency bands (Miller et al., 2008). This is because other frequency bands modulate up and down independently during different tasks and brain states, masking the broadband increase in power. The higher frequency components are more asynchronous, and therefore are not as subject to this masking effect (Hermes et al., 2017). Lower frequency bands, such as the alpha (8–12 Hz) and beta (13–30 Hz) band, are thought to represent pulsed inhibition that serves to gate and coordinate neuronal firing (Schalk, 2015). Therefore, analysis of broadband gamma activity reveals the local neuronal firing dynamics, while analysis of theta (4–8 Hz), alpha, and beta frequency regimes yields insight into the coordinating mechanisms across the brain.

The different oscillatory features discussed above have been explored for advancing our understanding of how different cortical regions function during motor movement and language function (Bouchard et al., 2013; Flint et al., 2017). Measurements of these signals during motor and speech imagery have been employed in ECoG brain–computer interfaces to drive end effectors such as computer cursors (Leuthardt et al., 2004, 2006a,b) and robotic arms (Hotson et al., 2016). Furthermore, non-motor regions can be used to drive ECoG-BCIs as well, illustrating the general utility of oscillatory band driven BCIs (Ramsey et al., 2006; Wilson et al., 2006).

## Current Clinical Uses of Direct Electrical Stimulation

### Functional Mapping for Epilepsy and Tumors

As detailed in the introduction, DES is frequently used both intraoperatively and during a patient’s stay at the hospital for functional mapping and identifying areas of cortex associated with important cognitive functions (Berger et al., 1989; Ojemann et al., 1989; Berger and Ojemann, 1992). These mapping procedures are done both for epilepsy surgery and tumor resections (Berger et al., 1989; Ojemann et al., 1989; Berger and Ojemann, 1992). Clinicians, using implanted ECoG electrodes or stimulators in the operating room, apply DES to various cortical and subcortical structures and pathways, and observe location dependent effects, including speech arrest in language regions, motor movements in motor cortex, and sensory percepts in somatosensory cortex. The results of these stimulation studies inform where the surgeons will plan to resect; for example, if the seizure focus is close to a language region, the surgeon and patient may decide the surgery is not worth the risk of a permanent language deficit.

### Deep Brain Stimulation

Deep brain stimulation is a prominent example of electrical stimulation of the brain. It is currently being used for therapeutic treatment of movement disorders [Parkinson’s disease (Bronstein et al., 2011) and Essential Tremor (Della Flora et al., 2010)], and is also being explored for treating psychiatric illnesses (post-traumatic stress disorder, depression, obsessive compulsive disorder, Tourette syndrome, Schrock et al., 2015) and epilepsy treatment. Traditionally, linear probes of cylindrical contacts are inserted into deep brain structures such as the globus pallidus internus (GPi), subthalamic nucleus (STN), or ventral intermediate nucleus of the thalamus (VIM). Following implantation, clinicians may either be guided by intraoperative CT imaging, or wake the patient up intraoperatively to test for adverse effects of stimulation on different contacts, using a monopolar (one stimulating electrode and a distant return electrode), bipolar (two similarly sized electrodes), or multipolar arrangement of electrodes for the steering of current.

Advances in BCI related to DBS have explored the use of closed loop DBS to trigger stimulation of deep brain structures in response to signals recorded from the surface of the cortex (Herron et al., 2017). Herron et al. used threshold crossing in the beta-band regime of recorded ECoG signals over motor cortex as a control decision to trigger DBS stimulation. This enables control of DBS stimulation solely through recorded neural signals. Besides potentially reducing the side-effects of open-loop stimulation, such closed-loop control of stimulation conserves power and helps extend the life of the DBS device, reducing the number of replacement surgeries needed over the life of a user. Adaptive DBS based on recordings in STN has been demonstrated to improve motor scores over traditional open-loop DBS (Little et al., 2013). In addition, primate models of Parkinson’s disease demonstrate that closed-loop DBS has a greater effect than open-loop DBS on akinesia and on the neuronal output in both cortical and subcortical structures (Rosin et al., 2011).

Finally, DBS is also being explored for the treatment of particular types of epilepsy. Partial onset seizures often spread through the circuitry of the basal ganglia, and therefore could be controlled using DBS strategies similar to those used for movement disorders (Halpern et al., 2008; Lega et al., 2010).

### Closed Loop Stimulation for Epilepsy

Closed loop stimulation to control seizures is currently clinically available to epilepsy patients through the Neuropace RNS system (Morrell, 2011; Lee et al., 2015). A neurosurgeon implants ECoG electrodes either on the cortical surface or in deeper structures near the putative seizure focus. If an impending seizure is detected, high frequency stimulation is triggered near the seizure focus to control the seizure. This is a demonstration of clinically effective and already implemented DES in an ECoG-BCI, where neural control signals are acquired in real time from the brain and used to trigger stimulation.

## Advantages of Des Relative to Other Stimulation Techniques

An advantage of DES relative to non-invasive electrical stimulation modalities is the delivery of much greater amounts of the applied current to neurons. During TES^2^, as much as 75% of the current is shunted through the scalp and the skull (Vöröslakos et al., 2018; Widge, 2018). This greatly blunts the efficacy of cortical stimulation, and suggests that some of the published results using TES are due to mechanisms other than direct neuronal excitation. In contrast, by directly stimulating the brain and bypassing the skull and scalp, DES delivers current to cortical structures more effectively. Although the currents applied during TES could be raised to a high enough level to reach a desired electric field strength at a target location in the brain, there would be potential off-target effects and skin damage due to the amount of current required, in contrast to DES through electrodes implanted precisely at the targeted site for this same electric field strength. This reinforces a large advantage of the DES relative to TES, which is the ability to place electrodes close to the target structures, and consequently minimize the amount of current passing through off-target structures.

Even with epidural and subdural stimulation, not all current reaches neurons in the cortex. Epidural stimulation results in current shunting by the dura (Wongsarnpigoon and Grill, 2008), while both epidural and subdural stimulation have some degree of cerebrospinal fluid (CSF) shunting depending on the characteristics of the CSF beneath or surrounding the electrodes (Wongsarnpigoon and Grill, 2008; Guler et al., 2018).

A factor in epidural and subdural stimulation is the presence of pain receptors within the dura which can be activated with dural stimulation (Wirth and Van Buren, 1971). However, previous clinical trials with epidural stimulation made no reports of dural pain with stimulation up to 6.5 mA and 250 μs pulse widths (Levy et al., 2008).

Transcranial magnetic stimulation has primarily been used to induce motor movements, rather than isolated sensory percepts (although phosphenes can often be produced via TMS, and tapping sensations and auditory clicks can accompany TMS) (Sliwinska et al., 2014). A method such as DES affords the ability to focally and specifically produce sensations that would not be achievable through TMS.

Additionally, traditional figure-8 TMS coils are currently unable to target cortical structures beyond 2–3 cm deep (Roth et al., 1991; Wagner et al., 2009). DES electrodes, on the other hand, can be physically placed in deeper regions of interest in order to elicit the desired stimulation effects. Another advantage of DES over TMS is the fact that the maximum of the electric field strength induced by TMS has to occur at the cortical surface rather than deeper structures (Heller and van Hulsteyn, 1992). This means that off-target effects in cortical layers near the surface are possible when targeting deeper structures. Even with more sophisticated coils, such as the H-coil, the maximum stimulation strength still occurs at the surface and greater depth of stimulation (4–6 cm) is achieved with a loss of focality (Zangen et al., 2005; Wagner et al., 2009). Although the field strength is greatest at the cortical surface for TMS, the orientation of neurons is a critical component in the activation of neurons, as both experimental and modeling work has shown that electric fields tangential to the sulcal walls can activate neurons oriented perpendicularly to them (Fox et al., 2004; Silva et al., 2008; Seo et al., 2016). Similarly, different layered pyramidal neurons are activated differently between the gyral crown and sulcus walls (Silva et al., 2008; Seo et al., 2016). This in total points to the complex physiologic effects of TMS, and the potential difficulties in activating groups of neurons both on the crown of the gyrus and within the sulcus together. A further disadvantage of TMS is that with current hardware, use outside of the lab is limited due to the bulky hardware and the need to maintain a precise spatial relationship between the coil and the head for stimulation.

The fact that DES electrodes can be placed near the deeper structures of interest is vital for the treatment of Parkinson’s and Essential Tremor through DBS. As these structures cannot currently be effectively stimulated through alternative methods such as TMS, effective clinical treatment relies upon DES via electrodes near the desired brain regions.

## Financial, Translational, Regulatory, and Ethical Concerns for Des in ECOG-BCIS

### Translational, Regulatory, and Financial Concerns

We expect early applications of ECoG-BCIs to leverage existing clinical devices. This has been a pathway forward for many prior medical devices. Advances in early DBS devices were based largely off of prior work in cardiac pacemaker and spinal cord stimulation devices (Coffey, 2009). We imagine a similar trajectory for DES in ECoG-BCIs. Preliminary use of DES has been enabled by investigational device exemptions (Harvey and Winstein, 2009). Further iterations of Medtronic DBS devices, such as the PC + S device, have been granted an investigational device exemption in research studies, and are improvements upon an already clinically approved device (Herron et al., 2017).

Whenever new technology is implemented for clinical treatment, a question of cost efficacy is raised. However, we suggest that ECoG-BCIs have the potential to be cost effective long-term devices if clinical efficacy is demonstrated, as illustrated by examples such as vagus nerve stimulators and DBS. Vagus nerve stimulation for epilepsy has been show to be effective long-term, and cost benefit analysis has shown that the cost of the treatment pays off within a 2 year period (Boon et al., 1999). Although it is not universally the case, DBS in general is thought to be cost effective, when looking at studies across European and North American Centers (Pereira et al., 2007). It has been noted that during the adoption of DBS large-volume hospitals had lower prices and superior short-term outcomes, which is something to be aware of in the translation of ECoG-BCIs into the clinic (Eskandar et al., 2009).

### Ethical Concerns

Ethical concerns are critical to address for any engineered device which is implanted in a patient. A previous review has explored some of the ethical concerns for BCIs (Klein and Ojemann, 2016), and we seek here to highlight some of the concerns which are particularly relevant to ECoG-BCIs with DES.

Articulating the potential risks and long-term requirements for an ECoG-BCI, particularly with DES, is essential for appropriate informed consent. Biologic risks such as infection, seizures, and tissue damage from stimulation (Cogan et al., 2016) are accompanied by technological concerns such as repeated surgeries for battery replacements, heating due to potential wireless charging, and lifetime electrode wear from repeated stimulation (Klein and Ojemann, 2016).

Privacy and security are another key aspect in implantable medical devices, particularly with any BCIs that communicate signals wirelessly or can be programed wirelessly. One can imagine situations where a stimulator could be set to either less than therapeutic levels or to unsafe levels, by malware transmitted to the ECoG-BCI device. Research efforts that build on current security and privacy protocols for medical devices are required to ensure neural signal security and protection against malevolent programing.

## Research Directions for Des in ECOG-BCI

We discuss various research directions for ECoG-BCIs, with an emphasis on future engineered applications. A previous review (Wander and Rao, 2014) has explored the use of brain–computer interfaces for investigating scientific questions in the nervous system. Further information on classical ECoG-BCIs and comparison to other types of BCIs can be found in Rao (2013).

### Sensory Feedback Through DES

One potential use of ECoG-based stimulation currently being explored is the restoration of sensory feedback for those suffering from disorders such as paralysis. There is a large clinical need, as it is estimated that 5.4 million Americans are living with paralysis, with an estimated 41.8% of people with paralysis unable to work (Christopher and Dana Reeve Foundation, 2013). The restoration of sensation is a priority for prosthetics users (Biddiss et al., 2007) as well as potential BCI end users such as individuals with paralysis (Anderson, 2004; Collinger et al., 2013). Sensory feedback to cortex would enhance the efficacy of a prosthetic arm to aid with independent tasks, or help an individual better interpret data from body mounted sensors. The lack of sensory feedback in many existing brain–computer interfaces (BCIs) may limit performance (Bensmaia and Miller, 2014; Delhaye et al., 2016). Indeed, integration of somatosensory feedback into BCIs has been demonstrated to improve task performance with BCIs (Suminski et al., 2010; Klaes et al., 2014; Dadarlat et al., 2015; Pistohl et al., 2015; Schiefer et al., 2016).

Prior work has shown that humans can respond to DES of the surface of the primary somatosensory (S1) cortex (Ray et al., 1999; Libet et al., 1964; Johnson et al., 2013; Hiremath et al., 2017), which results in an artificial sensory percept organized according to the standard somatotopy of cortex. Cronin et al. (2016) demonstrated that DES of S1 could be used by an individual in the absence of visual feedback to perform a motor task. Although these percepts would not be mistaken by the individuals for natural touch (Johnson et al., 2013; Cronin et al., 2016; Collins et al., 2017), they are useful for performing closed-loop BCI tasks. An open question is how using DES for feedback compares to a normal somatosensory pathway. One way of assessing this is through response times, which have recently been demonstrated to be slower for DES relative to natural touch (Caldwell et al., 2019). This speaks to the complex effects of stimulation and requires further exploration. Another key consideration for neuroprosthetic use is the embodiment of the prosthetic device. DES through ECoG has been shown to induce prosthetic hand ownership, suggesting that prostheses could be made to feel more natural as a result of DES (Collins et al., 2017).

With recent advances in materials and manufacturing, spatially smaller microECoG arrays are able to target smaller volumes of cortex. More targeted DES through microECoG grids allows higher spatial selectivity relative to larger clinical electrode grids (Hiremath et al., 2017; Lee et al., 2018), opening up the possibility of encoding more complex percepts compared to larger electrodes.

Although short term studies have demonstrated that these percepts induced by DES do not feel natural, the principles of neuroplasticity, which are prevalent in somatosensory cortex and other associated regions, and adaptation within the cortex (Miller and Weber, 2011; Weber et al., 2012; Thomson et al., 2013) will be relevant in the long-term implementation of DES in ECoG-BCIs for sensory restoration.

**FIGURE 2:**
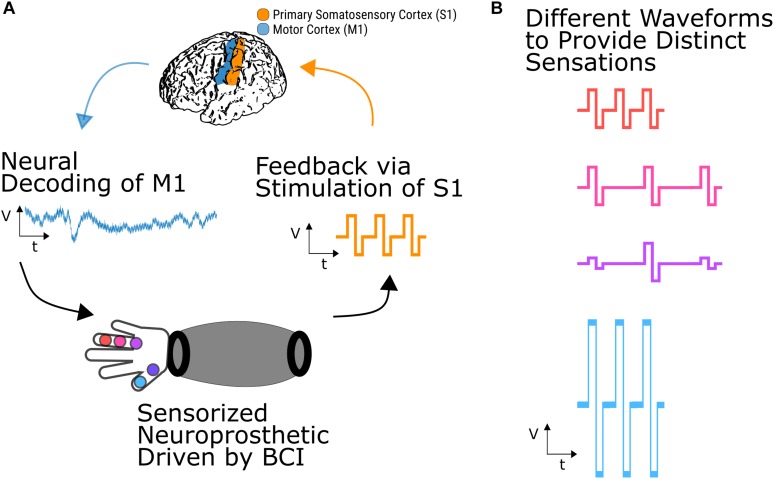
Somatosensory BCI with closed-loop stimulation. **(A)** Neural signals recorded from cortical regions such as primary motor cortex could be used to drive a sensorized prosthetic arm. Feedback about task performance or object manipulation could then be conveyed to the user by DES of primary somatosensory cortex. **(B)** Different stimulation parameters, such as amplitude, frequency, and carrier frequency shape, could convey different percepts which a user could learn to map to locations on the neuroprosthetic arm.

A BCI application with DES (Figure 2) could use signals from motor cortex to drive a sensorized prosthetic arm, which could provide feedback about the task via DES of primary somatosensory cortex (Figure 2A). Depending on the potential parameter space of discernible stimulation percepts, a user could learn to map physical contact locations on the prosthetic arm to distinct stimulation percepts (Figure 2B) providing feedback from external sensors directly to the brain The recent demonstrations of usable sensory signals in humans via DES brings us a step closer to such closed-loop human BCIs.

### Quantification of Cortical Connectivity

An additional application of DES is in quantifying cortical connectivity. DES of a cortical site can produce a cortico-cortical evoked potential (CCEP) at local and remote sites depending on the cortical area stimulated and the intensity of stimulation (Keller et al., 2014b). Studies have explored CCEPs in the context of different cortical networks, including language (Matsumoto et al., 2004) and motor regions (Matsumoto et al., 2006). The connections probed with CCEPs correspond well with known functional networks observed through fMRI as well as white matter pathways confirmed by diffusion tensor imaging (DTI) (Keller et al., 2014a). Such evoked potentials could have utility in BCI applications where depending on the presence or modulation of these CCEPs, an algorithmic decision could be made.

### Modification of Cortical Excitability and Induction of Plasticity

Another use for DES currently being explored is the induction of cortical plasticity. This refers to enhancement or other modification of connectivity between different cortical regions, which could aid in the recovery of individuals suffering from disrupted neuronal communication due to injuries such as stroke. To put the clinical need in perspective, there are millions of individuals worldwide who are disabled due to stroke. It is estimated that in the US alone for the year 2016, healthcare and economic costs related to stroke disability totaled $34 billion with stroke being a leading cause of serious long-term disability (CDC, 2015). 50–70% of stroke survivors reach functional independence, but 15–30% of survivors are permanently disabled (Lloyd-Jones et al., 2010). Therapies using targeted activity-dependent neuromodulation may help restore motor recovery (Harvey et al., 2009) but the biological effects of cortical stimulations are not well understood, and the parameters for potentially effective stimulation protocols need further development. Studies with smaller populations of neurons, animal models, and non-invasive stimulation may lend insight into the optimal protocols for plasticity induction.

A persistent theme in cortical connectivity is the idea of Hebbian plasticity, a type of synaptic plasticity first proposed by Hebb (1949): presynaptic firing of one neuron (site A) can strengthen the connection between it and a postsynaptic neuron (site B) that fires soon after A. Bi and Poo demonstrated a version of this plasticity rule, known as spike timing dependent plasticity (STDP), in rat hippocampal slice cells: consistent firing of a presynaptic cell (site A) within a time window of 20–30 ms before another postsynaptic cell (site B) led to a strengthened connection (LTP) from A to B, while B firing in a time window of 20–30 ms before A led to a weakened connection (LTD) (Bi and Poo, 1998). Both of these mechanisms were determined to be dependent on NMDA receptors.

These principles have been applied to induce plasticity in non-human primate (NHP) motor cortex (Jackson et al., 2006) and rodent rehabilitation experiments, where triggering stimulation in somatosensory cortex several milliseconds after premotor cortex firing in rats that suffered from damage to motor cortex resulted in increased functional performance (Guggenmos et al., 2013). Other work has explored the use of paired-pulse paradigms in NHPs to induce plasticity: where concurrent surface to depth stimulation at one site was consistently followed by stimulation at another site with a fixed time lag (Seeman et al., 2017). The optimal time lag for potentiation was found to be between 10–30 ms, with longer delays not resulting in potentiation. Only a fraction of the sites in this study were potentiated, and effects were often seen globally, illustrating the complex factors influencing cortical plasticity. A recent study in NHPs examined the timing of DES relative to the aggregate activity of neurons: DES delivered during beta oscillations during the depolarizing potential (negative peak as recorded through LFPs) caused potentiation of cortical connectivity, while DES delivered during the hyperpolarizing potential caused depression of cortical connectivity as assessed through cortically evoked potentials (Zanos et al., 2018).

Beyond work in animals, and importantly, for applications such as stroke rehabilitation, recent work has reported improvements in physiological measures of motor function with non-invasive stimulation such as movement triggered TMS compared to random TMS stimulation (Buetefisch et al., 2011). Adding further support to the importance of brain state dependent stimulation for rehabilitation is a recent study that demonstrated TMS delivery during movement-related beta-band (16–22 Hz) desynchronization caused a significant increase in corticospinal excitability, as evaluated through motor evoked potentials, lasting beyond the period of stimulation (Kraus et al., 2016).

Keller et al. (2018) demonstrated that repetitive 10 Hz DES using subdural electrodes induced both potentiation and suppression in different cortical sites, depending on the baseline network characteristics. This suggests that plasticity can indeed be modulated through DES in humans, and that individual patient models of connectivity may inform the optimal sites to target to either enhance or decrease connection strength.

**FIGURE 3:**
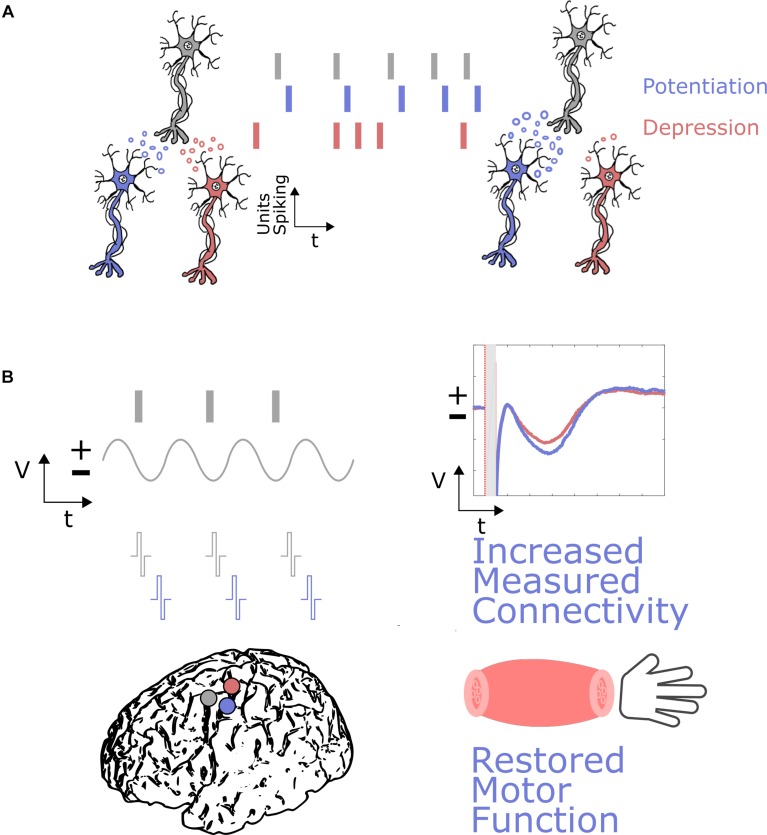
Neural plasticity induction through neuromodulation via DES in ECoG-BCIs. **(A)** The basic principles of neural plasticity involve the timing of activity between neurons resulting in the strengthening of connections, where potentiation occurs if the neurons fire with the appropriate timing in a causal manner, and depression occurs if neurons do not fire with the appropriate timing. **(B)** These principles could be used for neuromodulation through DES and ECoG-BCIs by stimulating near a particular damaged cortical region (purple), based on activity at a spared cortical region (gray). This activity could be a marker of neuronal firing, or a local field potential representing when neurons are more likely to be firing synchronously. Appropriately timed stimulation could then result in increased connectivity, measured through markers such as evoked potentials, and restored motor function relative to baseline. A damaged region not undergoing neuromodulation is shown in red, where evoked potentials are not positively modulated and motor function is not restored.

An example BCI application for neuromodulation (Figure 3) could include an oscillatory feature at a surface electrode, such as activity in the beta band or high gamma activity representing coordinated neuronal firing, driving stimulation at a damaged cortical region to enhance cortical connectivity and help restore motor function. This activity dependent stimulation could be similar to the activity-dependent DBS paradigms being explored (Herron et al., 2017). A more sophisticated approach, based on the concept of neural co-processors (Rao, 2019), could utilize artificial neural networks to map complex ECoG activity patterns at multiple recording sites to stimulation patterns at multiple stimulation sites to achieve goal-directed rehabilitation.

The combination of theoretical, animal, and human data discussed above suggests that activity-dependent DES is a promising approach to enhance and modify connectivity in humans, offering a new type of therapy for targeted restoration of function after neural injury. ECoG-BCIs are well-suited to acquiring and decoding appropriate control signals and when coupled with DES, can be used to influence cortical activity and induce activity-dependent plasticity.

## Limitations and Considerations

While ECoG based bi-directional BCIs offer several advantages over other types of BCIs, there are limitations and considerations that must be taken into account. For either subdural or epidural electrodes, neurosurgery is required. The size of the electrodes, relative to other invasive methods such as ICMS, results in larger population of neurons being targeted. Furthermore, there is no ability to target specific types of cells. Additionally, larger neurons with larger diameter axons are more likely to be activated by electrical stimulation (Tehovnik et al., 2006).

The developing field of optogenetics (Deisseroth, 2011; Yizhar et al., 2011) describes the use of genetic modification and optical methods to either activate or inactivate specific neurons *in vivo*. Optogenetics has been demonstrated to change functional connectivity in sensorimotor cortex in NHPs (Yazdan-Shahmorad et al., 2018). Although optogenetics may offer a more targeted approach to activating neurons, progress to humans may be slow due to the technique’s reliance on genetic modification of neurons.

Another current consideration when developing technologies and protocols to induce plasticity is our current lack of understanding of the mechanisms of plasticity induction (Feldman, 2012). Beyond the single neuron spiking level, plasticity is a complex phenomenon as discussed above, and in a human brain, the potential factors influencing plasticity can be complex and numerous. Optogenetics, with its ability to selectively target different populations of neurons will help provide critical insight into the mechanisms of plasticity.

Although DES may offer a promising approach to inducing plasticity, it has yet to be demonstrated to be unequivocally effective in a stroke model. Limited subgroups of stroke patients with residual motor function were shown to benefit from open-loop DES in the EVEREST trial, but other groups showed no benefit (Levy et al., 2016). As better animal models of stroke are developed (Sommer, 2017), one can hope to gain a better mechanistic understanding of how DES can be used for stroke rehabilitation, leading to optimized therapies for maximizing functional recovery following cortical injury.

The issue of particular patient subgroup benefit as discussed above speaks to the broader issue of patient variability. Due to anatomic or surgical variations, results from one group of subjects may not necessarily apply to another. Careful consideration of these individual factors will be important for future bidirectional ECoG-BCIs.

An additional consideration is the durability of electrodes with repeated stimulation. As mentioned in section “The Electrical-Neural Interface” above, charge transfer can occur through irreversible Faradaic reactions, where electrolysis occurs, and depending on the polarity of stimulation, either hydrogen gas or oxygen gas are the by products (Merrill et al., 2005). In this electrolytic window, accelerated corrosion and electrode damage can occur. Even below the voltage required for the electrolysis of water, detrimental byproducts such as the formation of metal chloride and hydrogen peroxide can occur, leading to electrode corrosion. Therefore, long-term use of stimulating ECoG electrodes will require careful selection of stimulation parameters and materials to minimize adverse effects. Relative to monophasic pulses, both charge balanced and imbalanced biphasic waveforms result in less electrode potential shift and accumulation of charge. Accumulation of charge during monophasic stimulation can result in additional undesirable Faradaic reactions, and the formation of reactive oxygen species which can cause tissue damage (Merrill et al., 2005). When comparing charge balanced and charge imbalanced biphasic waveforms, charge imbalanced waveforms have the advantage that at the end of each stimulation pulse, the electrode potential is closer to that of the open-circuit potential, resulting in less charge going to irreversible oxidation reactions (Merrill et al., 2005).

Beyond electrode damage, tissue damage induced by stimulation is a key consideration for long-term use of DES. The study of electrical stimulation through platinum electrodes in cats (McCreery et al., 1990) was used to define the Shannon equation (Shannon, 1992), which has been used frequently for assessing safe stimulation levels. Earlier research established a 30 μC/cm^2^ limit on the charge per phase of stimulation for macro-scale electrodes (in particular, DBS electrodes) (Kuncel and Grill, 2004), but tissue damage can occur above and below this threshold (Cogan et al., 2016). There are factors influencing whether or not tissue damage occurs that are not included in the Shannon equation, for example, the scale of the electrode (macro vs. micro), the current density, duty cycle, pulse frequency, and the uniformity of current distribution (Cogan et al., 2016). These complex factors will require further modeling and laboratory testing to establish what the appropriate stimulation parameters are to minimize tissue damage, particularly with the use of novel materials and stimulation patterns.

With penetrating microelectrodes (such as with the Utah array), there is a significant change in the electrode-tissue interface over time (Williams et al., 2007). In addition, stimulation can change the characteristics of the electrode-tissue interface. A recent study analyzing the impedance characteristics of DBS electrodes following implantation and stimulation has shown that DBS electrode impedance increases after implantation and decreases with clinically relevant stimulation (Lempka et al., 2009). Other work has shown that the stimulation parameters used affect the impedance measured for DBS electrodes (Wei and Grill, 2009). ECoG electrode impedance measurements from 191 persons implanted with the Neuropace RNS system, over a median time period of 802 days, did not reveal significant differences between stimulating and non-stimulating electrodes in peri-implant changes in impedance or impedance stability (Ryapolova-Webb et al., 2014). In this study, while there were statistically significant short-term changes in impedance following implantation, long-term impedances were stable. These results suggest that ECoG-BCIs with concurrent DES may prove viable as chronic implants.

## Enabling Technologies

### Materials and Electronics

Advances in materials science and electronics are enabling the creation of robust intracranial arrays with thousands of electrode contacts, with closer spacing than is currently used clinically. Current ECoG arrays based on silicone and platinum have been extended to microECoG arrays (Chao et al., 2010). Further reductions in electrode diameter and increases in array density are enabled by micromanufacturing techniques, and in particular, microelectricomechanical systems (MEMS) technologies. Platinum electrodes and polyimide foil substrates similarly have been patterned using micromachining, allowing for electrode contact diameters of 1 mm with electrode spacings between 2 and 3 mm (Rubehn et al., 2009). Through these MEMS technologies, electrode arrays with tighter spacings and smaller diameters can be constructed and placed across large regions of cortex and within sulci (Fukushima et al., 2014). Fukushima et al. (2014) created an array with 0.8 mm diameter electrodes and 1.8 mm spacing.

MicroECoG arrays have recently been used to resolve finer features of cortical activity, particularly in the broadband gamma range, for measurement of phonetic features in single electrodes (Mesgarani et al., 2014). Arrays with electrode diameters of 0.87 mm and spacings of 1.68 mm have resolved cortical activity patterns with response peaks less than the standard clinical spacing of 1 cm apart, pointing to the advantages seen with smaller electrode arrays (Wang et al., 2017). Novel, thin film MEMS arrays are being implanted in humans (Muller et al., 2016), illustrating the translation of these materials and manufacturing techniques to humans. The ability to place more electrodes within a given area could allow for finer patterning of stimulation.

Advances in materials science are enabling electrodes and arrays made of other materials, such as glassy carbon (Kassegne et al., 2015; Goshi et al., 2018). Glassy carbon electrodes have higher charge injection capacities (CIC, which is the amount of charge that can be injected before irreversible chemical reactions take place) than traditional platinum electrodes, and require less stimulation current to activate neurons (Kassegne et al., 2015).

Combinations of ECoG and penetrating electrode arrays are also being constructed for recording and stimulating both surface and deeper structures simultaneously (Orsborn et al., 2015; Goshi et al., 2018; Kleinbart et al., 2018). Currently being used in animal models, such arrays will open the door to a better understanding of network-wide and across cortex effects of stimulation.

### Computational Modeling

Computational modeling may help inform the design of DES targeting strategies by delineating which areas of cortex are activated during different polarities of stimulation, and which combinations of electrodes may prove effective. For example, a computational model of subdural cortical stimulation based on anisotropy estimates from DTI revealed that neurons deeper in the cortex are activated more during cathodal subdural stimulation, while those in the wider crown are activated during anodal stimulation (Seo et al., 2015). The influence of anisotropy on neuronal excitation from DES illustrates the importance of detailed, accurate anatomy for understanding and predicting the effects of DES.

A multicompartment computational model for subdural DES illustrated the effect of the neuronal structure, size, and orientation on activation thresholds (Kudela and Anderson, 2015). In the model, the specific parameters of stimulation and structure of the axons influenced the presynaptic terminal activation.

The combination of FEM and patient specific CT and MRI imaging has enabled the optimization of current delivery to various cortical regions depending on desired parameters, such as minimizing current density in particular regions (Guler et al., 2018). Combining individual patient MRIs with accurate computational models of how neurons are activated will allow precise DES targeting, with potentially fewer off-target effects.

The DBS field is replete with examples of new modeling techniques to optimize stimulation of deep cortical targets. These advances could carry over more generally to DES in ECoG-BCIs. Patient specific models of the volume of tissue activated (VTA) enable better understanding of the effects of stimulation at various locations in a given individual (Butson et al., 2007). With the advent of electrodes with many contacts and different geometries, an open question is how to best target the region of interest. Recent algorithmic advances combine electrodes with different contact geometries, including cylindrical and directional leads, and patient specific models, including tissue anisotropy, to best target the sub-thalamic nucleus (STN) (Anderson et al., 2018). A multi-objective particle swarm optimization technique to select a combination of stimulation electrodes was found to be more effective than a single monopolar electrode in targeting the desired efferents from the STN (Peña et al., 2018). As ECoG electrodes become smaller and more numerous, algorithmic techniques such as the ones described above and more advanced ones based on artificial neural networks (Rao, 2019) would enable precisely targeted DES with the right combination of electrodes.

### Concurrent Recording and Stimulation

In any closed-loop application involving concurrent stimulation and recording, the electrical artifact due to stimulation is many orders of magnitude greater than the neural signals being recorded. Disentangling the volume conduction of the stimulation pulse from the neural responses is a topic of active research. Different approaches have been used for handling artifacts, ranging from hardware approaches to mitigate artifacts before signal acquisition to post-processing techniques to minimize artifacts after the signals have been acquired.

An example system manufactured with CMOS technology enables both common mode and differential real time artifact cancelation (Smith et al., 2017). In combination with this, new CMOS stimulator front-ends are being developed which could allow for more scalable, integrated BCI devices with wireless, signal processing, and stimulator blocks (Pepin et al., 2016). Advances in this area will permit a better understanding of how the brain responds to electrical stimulation, as well as permit more complex closed loop applications (Zhou et al., 2018a) where neural activity in close temporal and spatial relation to the site of stimulation can be integrated into the control system.

Recent technology development in industry for simultaneous stimulation and recording in DBS applications both illustrates widespread interest in the development of concurrent stimulation and recording devices, and suggests potential combined hardware and software solutions for ECoG-BCIs (Stanslaski et al., 2012; Herron et al., 2018). These techniques include careful consideration of the stimulation and recording configuration to mitigate the measured artifact, front-end filtering, heterodyning to minimize stimulation harmonics in neural frequency bands of interest, and selection of stimulation parameters to aid in the separation of neural signals from stimulation artifacts (Stanslaski et al., 2012). Medtronic’s Summit RC + S system extends the previously mentioned approaches to simultaneous stimulation and recording, and further includes oversampling to reduce noise in the signal bands of interest, decimators designed to filter out higher-order harmonics from stimulation, as well as options to only suggest sense-friendly stimulation parameters to the researcher or clinician (Herron et al., 2018). Such techniques could be applied more broadly to include ECoG-BCI systems with DES.

### Wireless Technologies

Recent advances in hardware have allowed both real time artifact cancelation and wireless communication with 128 channels of local field potential recording in NHPs (Zhou et al., 2018b). Other implantable devices with microelectrode arrays in NHP model have included wireless charging and data transfer capabilities (Borton et al., 2013), which are critical for an out-of-hospital device. The development of wireless technologies, as well as real time simultaneous stimulation and recording techniques, opens the door to explorations of the neural basis of naturalistic behavior and long-term effects of closed-loop stimulation. Recent work in non-human primates has demonstrated both wireless recording and stimulation of motor regions over a 6 months time period, with no observed neurological or behavioral consequences (Romanelli et al., 2018). This points to the future translatability of wireless long-term ECoG implants with both recording and stimulation.

## Conclusion

Direct electrical stimulation of the human brain is currently used clinically for functional mapping, as well as therapeutic treatment of disorders such as epilepsy and movement disorders. In this article, we have explored DES can also be used as a new modality for providing input to cortex in electrocorticographic (ECoG) brain computer interfaces (BCIs). DES offers distinct advantages over other stimulation modalities such as TES and TMS by virtue of delivering electrical stimulation directly to the brain. We discussed some of the barriers for DES translation to ECoG-BCIs, and highlighted the progress being made in the use of DES for restoration of somatosensation and induction of cortical plasticity for targeted rehabilitation in stroke. We also have reviewed how advances in technology, including new materials for electrode design, manufacturing techniques for smaller electrode arrays, and computational modeling for tailoring stimulation to the patient’s needs offer opportunities for radically expanding the applications of DES in bi-directional ECoG-BCIs for restoring neurological function.

## Author Contributions

DC, JO, and RR planned the study and conducted some of the research on the topic of sensation induced by electrocorticographic stimulation described in this manuscript. DC wrote the first draft of the manuscript. DC, JO, and RR edited the draft and finalized the manuscript.

## Conflict of Interest Statement

The authors declare that the research was conducted in the absence of any commercial or financial relationships that could be construed as a potential conflict of interest.

## Footnotes

1 We discuss advances in research-grade microECoG electrodes with much smaller electrode contact size and tighter spacing in the section “Enabling Technologies – Materials and Electronics.”

2 We use this term to encompass transcranial direct current stimulation (tDCS) and transcranial alternating current stimulation or (tACS).
